# Immune Modulation Properties of Zoledronic Acid on TcRγδ T-Lymphocytes After TcRαβ/CD19-Depleted Haploidentical Stem Cell Transplantation: An analysis on 46 Pediatric Patients Affected by Acute Leukemia

**DOI:** 10.3389/fimmu.2020.00699

**Published:** 2020-05-12

**Authors:** Pietro Merli, Mattia Algeri, Federica Galaverna, Giuseppe Maria Milano, Valentina Bertaina, Simone Biagini, Elia Girolami, Giuseppe Palumbo, Matilde Sinibaldi, Marco Becilli, Giovanna Leone, Emilia Boccieri, Lavinia Grapulin, Stefania Gaspari, Irma Airoldi, Luisa Strocchio, Daria Pagliara, Franco Locatelli

**Affiliations:** ^1^Department of Pediatric Hematology and Oncology and of Cell and Gene Therapy, Scientific Institute for Research and Healthcare (IRCCS), Bambino Gesù Childrens' Hospital, Rome, Italy; ^2^Transfusion Unit, Department of Laboratories, Scientific Institute for Research and Healthcare (IRCCS), Bambino Gesù Childrens' Hospital, Rome, Italy; ^3^Department of Radiology and Radiotherapy, Sapienza University, Rome, Italy; ^4^Stem Cell Laboratory and Cell Therapy Center, Giannina Gaslini Institute (IRCCS), Genoa, Italy; ^5^Sapienza, University of Rome, Rome, Italy

**Keywords:** TcRγδ+ lymphocytes, zoledronic acid, TcRαβ/CD19 cell depleted haploidentical stem cell transplantation, acute leukemia, children

## Abstract

TcRαβ/CD19-cell depleted HLA-haploidentical hematopoietic stem cell transplantation (haplo-HSCT) represents a promising new platform for children affected by acute leukemia in need of an allograft and lacking a matched donor, disease recurrence being the main cause of treatment failure. The use of zoledronic acid to enhance TcRγδ+ lymphocyte function after TcRαβ/CD19-cell depleted haplo-HSCT was tested in an open-label, feasibility, proof-of-principle study. Forty-six children affected by high-risk acute leukemia underwent haplo-HSCT after removal of TcRαβ+ and CD19+ B lymphocytes. No post-transplant pharmacological graft-versus-host disease (GvHD) prophylaxis was given. Zoledronic acid was administered monthly at a dose of 0.05 mg/kg/dose (maximum dose 4 mg), starting from day +20 after transplantation. A total of 139 infusions were administered, with a mean of 3 infusions per patient. No severe adverse event was observed. Common side effects were represented by asymptomatic hypocalcemia and acute phase reactions (including fever, chills, malaise, and/or arthralgia) within 24–48 h from zoledronic acid infusion. The cumulative incidence of acute and chronic GvHD was 17.3% (all grade I-II) and 4.8% (all limited), respectively. Patients given 3 or more infusions of zoledronic acid had a lower incidence of both acute GvHD (8.8 vs. 41.6%, *p* = 0.015) and chronic GvHD (0 vs. 22.2%, *p* = 0.006). Transplant-related mortality (TRM) and relapse incidence at 3 years were 4.3 and 30.4%, respectively. Patients receiving repeated infusions of zoledronic acid had a lower TRM as compared to those receiving 1 or 2 administration of the drug (0 vs. 16.7%, *p* = 0.01). Five-year overall survival (OS) and disease-free survival (DFS) for the whole cohort were 67.2 and 65.2%, respectively, with a trend toward a better OS for patients receiving 3 or more infusions (73.1 vs. 50.0%, *p* = 0.05). The probability of GvHD/relapse-free survival was significantly worse in patients receiving 1–2 infusions of zoledonic acid than in those given ≥3 infusions (33.3 vs. 70.6%, respectively, *p* = 0.006). Multivariable analysis showed an independent positive effect on outcome given by repeated infusions of zoledronic acid (HR 0.27, *p* = 0.03). These data indicate that the use of zoledronic acid after TcRαβ/CD19-cell depleted haploHSCT is safe and may result in a lower incidence of acute GvHD, chronic GvHD, and TRM.

## Introduction

Hematopoietic stem cell transplantation (HSCT) is, so far, the best curative option for a number of malignant disorders (1, 2). However, up to 30% of patients lack both a HLA-identical sibling or an alternative donor [i.e., HLA-Matched Unrelated Donor (MUD) or Unrelated Donor Umbilical Cord Blood (UD-UCB)] (3). Thus, HSCT from an HLA-haploidentical relative (haplo-HSCT) represents a very promising option, since it owns particular features, including applicability for virtually all patients, choice of best donor from a panel of potential candidates, immediate accessibility to the transplant procedure, and easy access to donors in case adoptive cell therapies as are required after transplantation (3). However, compared to other types of allograft, haplo-HSCT has been hampered by a delayed immune reconstitution (4, 5), which, in turn, influences the risk of both post-transplant infections and, most importantly, relapse incidence (6, 7). Since it has been demonstrated that specific innate immunity cell subpopulations [e.g., NK (8) and TcRγδ T cells (9)] may influence transplant outcome, we and other groups recently developed a new method of graft manipulation (i.e., TcRαβ/CD19 negative selective depletion) (10–13), which allows to retain large numbers of mature, ready-to-kill effector cells, namely NK and TcRγδ lymphocytes, in the final product. TcRγδ cells are a non-alloreactive, “innate-like,” T lymphocyte subpopulation (normally accounting for 1–10% of circulating T lymphocytes) capable of recognizing targets in an MHC-independent manner (through several activating receptors, like γδ-TcR, NKG2D, and TLRs) and displaying different functions, including anti-infective and anti-tumor activity (14, 15). We have recently reported the largest pediatric cohort receiving this kind of transplant, clearly showing that this approach represents a suitable alternative for children affected by acute leukemia, with outcomes comparable to those of HLA-matched donor HSCT recipients (13).

Preclinical data showed that bisphosphonates, such as pamidronate and zoledronate, commonly used to treat bone diseases and hypercalcemia in multiple myeloma, can mediate the improvement of TcRγδ-mediated tumor cells killing capacity (16, 17). Such an effect is obtained thanks to the activation of a particular subset of TcRγδ cells, through the accumulation of phosphoantigens (18) and, to a lesser extent, the sensitization of tumor target cells (19).

In an attempt to reduce the relapse incidence and the risk of severe infections in high risk-patients given TcRαβ/CD19-cell depleted haploHSCT, we explored the administration of zoledronic acid in the post-transplant period (20). We previously reported detailed cytofluorimetric, functional, and proteomic analysis of TcRγδ cells of patients treated with this strategy, showing that zoledronate administration promotes γδ T-cell differentiation and cytotoxicity (20). Here we report the long-term outcomes of 46 patients treated with zoledronic acid after TcRαβ/CD19-depleted haploHSCT.

## Patients and Methods

### Patients

Patients aged 0.3 to 21 years, affected by acute leukemia, who were in need of an allograft while lacking an HLA-matched related or unrelated donor between January 1st, 2013 and September 26th, 2016 at IRCCS Ospedale Pediatrico Bambino Gesù in Rome, were considered eligible. All patients or legal guardians provided written informed consent, and the research was conducted under a Hospital Ethical Committee- approved protocol, in accordance with the Declaration of Helsinki.

### Transplantation Procedure

All the 46 enrolled patients received a fully myeloablative conditioning regimen, which was based on the use of total body irradiation (TBI) in 37 (80%) children and chemo-based in 9 patients (20%) (details are reported in the Supplementary Material). Pre-transplantation rabbit anti-T lymphocyte globulin (ATLG, Grafalon®, Neovii) was administered, at a dose of 4 mg/kg/day for 3 consecutive days (days−5 to−3), in order to prevent both graft failure and graft-versus-host disease (GvHD), through *in vivo* T-cell depletion and/or modulation of bidirectional alloreactivity. On day−1, children were also given rituximab 200 mg/m^2^ for *in vivo* donor and recipient B-cell depletion to reduce the risk of Epstein-Barr virus (EBV)-related post-transplantation lymphoproliferative disorders (PTLD). No patient was given any post-transplant pharmacologic GvHD prophylaxis.

The donor was chosen according to immune-genetic criteria, giving priority to natural killer (NK) alloreactivity (evaluated according to the killer immunoglobulin-like receptor (KIR)-KIR ligand model), NK cell B haplotype, and higher B content, as previously described (10, 21). The donor was a parent for all patients but one, who was transplanted from her HLA-haploidentical brother. Granulocyte colony-stimulating factor (G-CSF) at a dose of 10–12 μg/kg/day was administered by subcutaneous injection to all donors to mobilize in peripheral blood hematopoietic stem cells from day−5 until leukapheresis (day−1). Ten donors (21.7%) with circulating CD34+ cell count <0.04 x 10^9^/L on day−2 also received a single-dose of plerixafor (240 μg/kg) 6–9 h before cell collection. Graft manipulation was performed using the CliniMACS device as previously described (22).

### Zoledronic Acid Administration

Zoledronic acid was administered monthly at a dose of 0.05 mg/kg/dose (maximal single dose 4 mg) over 1 h, starting after: i) achievement of stable donor engraftment, and ii) at least day +20 from transplantation. The dose was based on literature data about zoledronate use in pediatric bone diseases (23). Since this was an open-label, feasibility, proof-of-principle study, the number of scheduled doses was not fixed; patients continued to receive monthly infusions of up to 5 consecutive doses, unless an event (i.e., side effects related to the drug, disease relapse, severe infections, hospitalization for any cause, patient/parents refusal) occurred. We opted to administer multiple infusions of zoledronic acid, based on current literature data indicating that *in vivo* activation of TcRγδ T-cells in response to the drug is a transient phenomenon (24). Oral calcitriol, together with calcium supplementation, was administered for 7–10 days after zoledronate infusion, in order to prevent/treat hypocalcemia. Zoledronic acid was administered either in the inpatient or in the outpatient unit.

### Statistical Analysis

Quantitative variables were reported as median value and range; categorical variables were expressed as absolute value and percentage. Clinical characteristics of patients were compared using the Chi-square test or Fisher's exact test for categorical variables, while the Mann-Whitney rank sum test or the Student's *T*-test were used for continuous variables as appropriate (25). The time to neutrophil engraftment was defined as time from HSCT to the first of 3 consecutive days with an absolute neutrophil count equal to or greater than 0.5 × 10^9^ per liter, and the time to platelet engraftment as time from HSCT to the first of 7 consecutive days with an unsupported platelet count equal to or greater than 20 × 10^9^ per liter.

Patients surviving for more than 7 and 100 days after transplantation were considered evaluable for acute and chronic GvHD (aGvHD, cGvHD) occurrence, respectively. Severity of aGvHD and cGvHD was assessed according to Glucksberg and Shulman criteria (26, 27).

Overall survival (OS), disease-free survival (DFS), GvHD/relapse-free survival (GRFS), transplant-related mortality (TRM), aGvHD, cGvHD, and relapse incidence (RI) were estimated from the date of transplantation to the date of an event or last follow-up. Probabilities of OS, DFS, and GRFS were calculated according to the Kaplan and Meier method (28). TRM, aGvHD, cGvHD, and RI were calculated as cumulative incidence curves in order to adjust the estimates for the appropriate competing risks (29). All results were expressed as probability or cumulative incidence (%) and 95% confidence interval (95% CI) (30).

The significance of differences between OS, DFS, and GRFS was estimated by the log–rank test (Mantel–Cox), while Gray's test was used to assess, in univariate analyses, differences between cumulative incidences (31). Multivariate analysis was performed using the Cox proportional hazard regression model (30). *P*-values < 0.05 were considered to be statistically significant.

Statistical analysis was performed using EZR version 1.32 (Saitama Medical Centre, Jichi Medical University), which is a graphical user interface for R (The R Foundation for Statistical Computing, Vienna, Austria) (32).

## Results

### Patients

Forty-six patients affected by acute leukemia received a TcRαβ/CD19-cell depleted peripheral blood stem cell allograft from an HLA-haploidentical relative in the study period at IRCCS Ospedale Pediatrico Bambino Gesù, Rome. Table 1 reports patient demographic and disease characteristics, and transplant details. Twenty-six patients had B-cell precursor acute lymphoblastic leukemia (BCP-ALL), 7 T-cell lineage ALL (T-ALL), and 11 acute myeloid leukemia (AML), while 2 patients were affected by mixed phenotype acute leukemia (MPAL). Seven patients had previously received a first HSCT. Two patients were not in morphological complete remission at time of transplantation.

**Table 1:** Patient demographic, disease characteristics, and transplant details.

	**Number**	**Percentage (%)**	**Median**	**Range**
**Total**	46	100		
**Gender**				
Male	33	72		
Female	13	28		
**Age at diagnosis, years**			9.2	0.6–22
**Age at transplant, years**			10.9	1–22.2
**Disease**				
BCP-ALL	26	57		
T-ALL	7	15		
AML	11	24		
MPAL	2	4		
**Phase of disease**				
CR1	13	28		
CR2	23	50		
>CR2	8	18		
Active disease	2	4		
**Genetic abnormalities**				
t(9;22)	2	4		
Complex karyotype	2	4		
FLT3-ITD	2	4		
Cytogenetic abnormalities involving 11q23^*^	3	7		
**Previous HSCT**	7	15		
**Conditioning regimen**				
TBI-based	37	80		
Chemo-based	9	20		
**Cell dose infused**				
CD34+ × 10^6^/kg			15.4	6.5–40.6
TCRαβ+ × 10^6^/kg			0.04	0.001–0.099
TCRγδ+ × 10^6^/kg			7.7	0.8–42.7
NK+ × 10^6^/kg			23.3	2.0–141.6
CD20+ × 10^6^/kg			0.03	0.001–0.18
**Donor Characteristics**				
Age (years)			39	21–56
Type of donor				
Mother	27	59		
Father	18	39		
Brother	1	2		
Sex mismatch (F->M)	23	50		
NK alloreactivity (KIR-KIR-L model)	19	41		
KIR genotype B/X	36	78		

* *1 case each of t(6;11), t(9;11) and t(10;11)*.

### Graft Composition and Hematopoietic Recovery

All children received <1 × 10^5^ TcRαβ cells per kg of recipient body weight (median 0.04 × 10^6^/kg; range 0.001–0.099), with a median Log depletion of 3.93 (range 3.42–5.42), while the median number of TcRγδ cells infused was 7.7 × 10^6^ per kg (range 0.8–42.7) (details of graft composition are reported in Table 1). The median number of infused CD34+ cells per kg was 15.4 × 10^6^ (range 6.5–40.6) (median CD34+ stem cell recovery 91.25%; range 71.67–100), while that of B cells was 0.03 × 10^6^/kg (range 0.001–0.18) (median Log depletion of 3.42; range 2.93–4.50).

All patients achieved engraftment; the median time to neutrophil engraftment was 14.5 days (range 8–21), whereas the median time to platelet recovery was 11 days (range 8–27). Monitoring of donor-recipient chimerism performed to hypervariable regions of human DNA confirmed the engraftment of donor hematopoiesis in all patients.

### Zoledronic Acid Infusions and Adverse Reactions

The 46 patients received a total of 139 zoledronic acid infusions, with a mean of 3 infusions per patient (range 1–6). Specifically, 4 patients received a single infusion, 8 patients received 2 infusions, 20 were given 3 infusions, 12 received 4 infusions, while one patient each received 5 and 6 infusions (because of physician decision, based on good tolerability and positive effect on bone metabolism parameters). Treatment started at a median of 39 days (range 20–79). Ten patients (21.7%) experienced an acute phase reaction, including fever, chills, malaise, arthralgia, and/or transient skin rash, within 24–48 h after the first administration. Five patients (10.8%) had a transient decrease in the WBC or platelet count. All patients experienced a reduction of calcium serum levels; however, only one episode of symptomatic hypocalcemia (at first administration) occurred and was rapidly corrected with parenteral calcium supplementation. One patient (2%) presented asymptomatic hypokaliemia. No other adverse event was recorded; in particular, we did not observe any case of osteonecrosis.

### TcRγδ T-Cells After Zoledronic Acid Infusion

**Figure 1:**
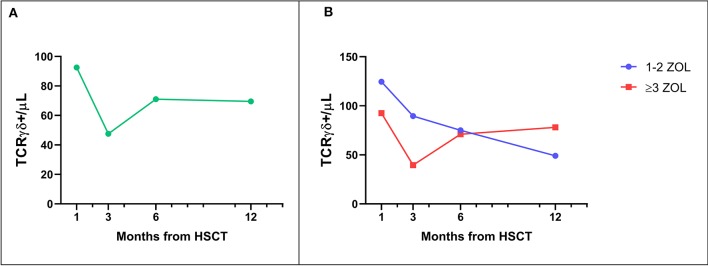
**(A)** Median number of TcRγδ T-cells in the peripheral blood of patients receiving zoledronic acid after TcRαβ/CD19-depleted haploHSCT. **(B)** Median number of TcRγδ T-cells in the peripheral blood of patients receiving either 1–2 or 3–6 infusions of zoledronic acid (ZOL).

As already reported (13, 16), TcRγδ T-cells represented the main T cell population in the first weeks after TcRαβ/CD19-depleted haploidentical HSCT. This predominance of TcRγδ T-cells was observed also in zoledronate-treated patients. Subsequently, TcRαβ T-lymphocytes progressively increased over time, exceeding the percentage of TcRγδ T-cells before the third month after transplantation (data not shown). Interestingly, as already shown (20), in patients given zoledronic acid a progressive decrease in the percentage and absolute number of TcRγδ T-cells was observed, thus suggesting that repeated infusions of zoledronic acid do not induce persistence of this cell subset (Figure 1A). No significant difference in the number of circulating TcRγδ T-cells between patients receiving ≥3 infusions of zoledronic acid as compared to those given 1 or 2 infusion was found, although there was a trend toward a higher reduction at 3 months after HSCT in the first group (Figure 1B).

### Graft-*versus*-Host Disease

**Figure 2:**
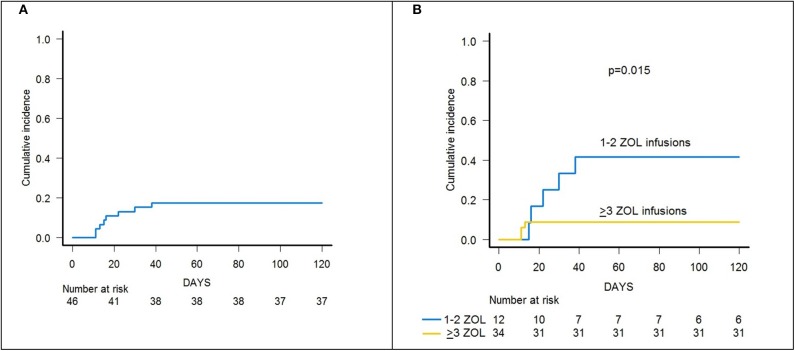
**(A)** Cumulative incidence of acute GvHD (all grades) of the whole cohort of 46 patients. **(B)** Cumulative incidence of acute GvHD (all grades) in patients receiving either 1–2 or 3–6 infusions of zoledronic acid (ZOL).

**Figure 3:**
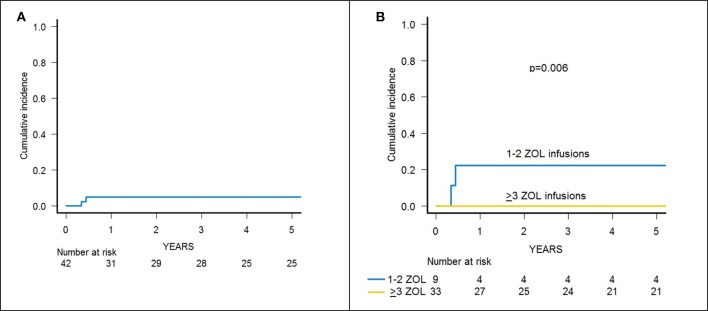
**(A)** Cumulative incidence of chronic GvHD of the whole cohort of 46 patients. **(B)** Cumulative incidence of chronic GvHD in patients receiving either 1–2 or 3–6 infusions of zoledronic acid (ZOL).

Skin-only aGvHD was observed in 8 children after zoledronic acid infusion, with 3 patients experiencing overall grade I and 5 patient developing grade II aGvHD, the cumulative incidence of aGvHD being 17.3% (95% confidence interval (CI) 5.6–27.6) (Figure 2A). Grade I aGvHD was treated with sole topical steroid application, while patients with grade II aGvHD received systemic corticosteroids with or without extracorporeal photopheresis (ECP), with complete resolution in all cases. Two out of the 42 evaluable patients developed limited cGvHD, with a cumulative incidence of 4.8% (95% CI 0–11.2) (Figure 3A).

Notably, patients given 3 or more infusions of zoledronate had a lower incidence of both acute and chronic GvHD. In detail, patients receiving either ≥3 doses or 1–2 doses of zoledronic acid had a cumulative incidence of aGvHD of 8.8% (95% CI 0–17.9) and 41.6% (95% CI 5.9–63.8), respectively (*p* = 0.015) (Figure 2B).

Since the 2 patients experiencing cGvHD received <3 infusions of zoledronic acid, the cumulative incidence of cGvHD in patients receiving 3 or more infusions of zoledronate compared to those who received 1 or 2 infusions was 0 and 22.2% (95% CI 0–45.1), respectively (*p* = 0.006) (Figure 3B).

### Infections

In terms of early virus reactivation, cytomegalovirus (CMV) DNAemia was recorded in 15 patients, with a cumulative incidence of 30.4% (95% CI 15.7–42.5). However, none progressed to CMV disease thanks to the administration of pre-emptive treatment with ganciclovir. Regarding EBV, no EBV-associated PTLD occurred in our cohort. Nine patients experienced Adenovirus infection (cumulative incidence 19.5%; 95% CI 7.2–30.2); in one case the infection was not controlled by pharmacological therapy, leading to a disseminated infection that ultimately resulted in the patient's death. Three patients developed bacterial infection (2 Gram-positive and 1 Gram-negative sepsis) (cumulative incidence 6.5%; 95% CI 0–13.4), controlled by large-spectrum antibiotic therapy. Finally, one patient suffered from a fatal *Aspergillus flavus* infection. The number of zoledronic acid infusion did not affect the incidence of viral, bacterial, or fungal infection. However, there was a trend toward a lower incidence of CMV infection in patients given 3–6 infusions (26.4 vs. 41.6% for patient receiving less administrations), but the difference was not statistically significant.

### Relapse and Transplant-Related Mortality

Fourteen patients relapsed at a median time of 194 days after HSCT (range 81–1,081), the cumulative incidence of relapse being 30.4% (95% CI 17.8–44.1). It did not differ between patients receiving 1–2 infusions of zoledronic acid and patients receiving 3–6 administrations (33.3 vs. 29.4%, *p* = n.s.), although there was a trend toward reduction of relapse for patients receiving 4–6 zoledronate doses as compared to those receiving 1–3 infusions (37.5 vs. 14.3%, *p* = 0.11) (Supplementary Figure 2). Time to first zoledronic acid infusion was comparable between relapsed and disease-free patients (median 39 vs. 42.5 days, *p* = n.s.). Moreover, time to relapse was comparable between relapsed patients receiving 1–2 zoledronic acid infusions and those receiving 3–6 administrations. Finally, the proportion of children receiving 3–6 infusions was comparable between the subgroup of patients in first complete remission (CR) and those in second CR/other disease *status* (*p* = n.s.). As already reported (13), the use of TBI in the conditioning regimen was associated with a reduced incidence of relapse (21.6 vs. 66.7%, *p* = 0.01).

**Figure 4:**
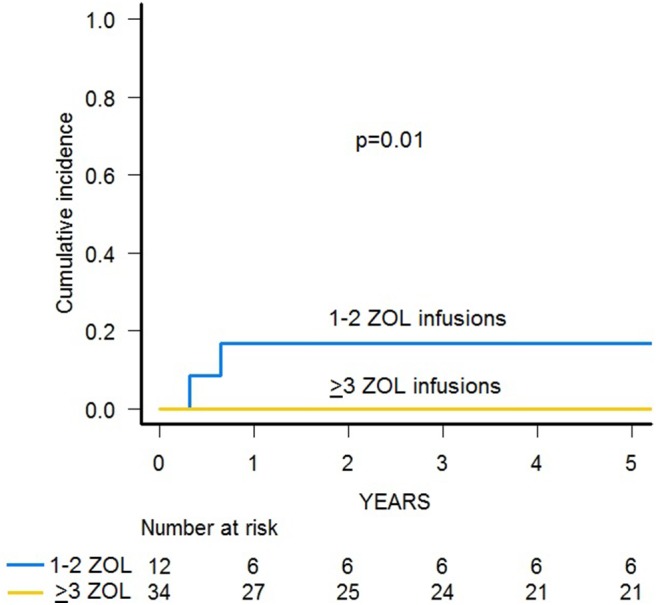
TRM of patients receiving either 1–2 or 3–6 infusions of zoledronic acid (ZOL).

Since two patients died due to infections, the 3-year cumulative incidence of TRM was 4.3% (95% CI 0.8–13.2) (Supplementary Figure 1). A significantly lower TRM was observed for patients who received 3–6 infusions of zoledronic acid in comparison with those given 2 or less administrations (0 vs. 16.7%, *p* = 0.01) (Figure 4).

### Survival Outcomes

**Figure 5:**
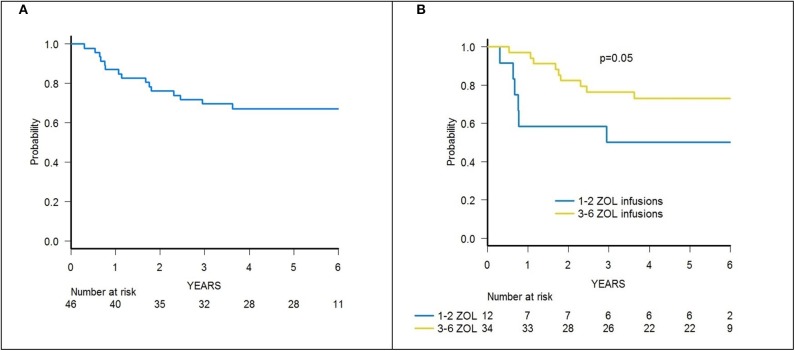
**(A)** OS of the whole cohort of 46 patients. **(B)** OS of patients receiving either 1–2 or 3–6 infusions of zoledronic acid (ZOL).

**Figure 6:**
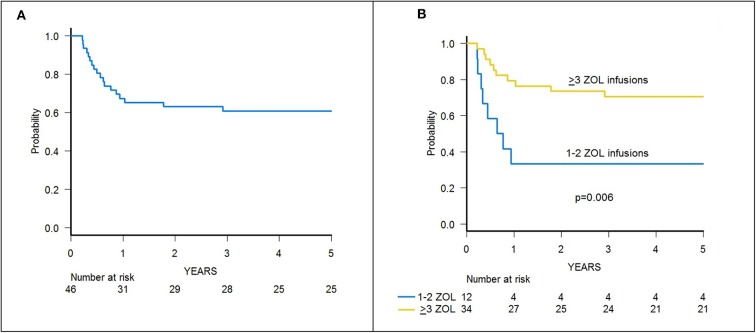
**(A)** GRFS of the whole cohort of 46 patients. **(B)** GRFS of patients receiving either 1–2 or ≥3 infusions of zoledronic acid (ZOL).

**Figure 7:**
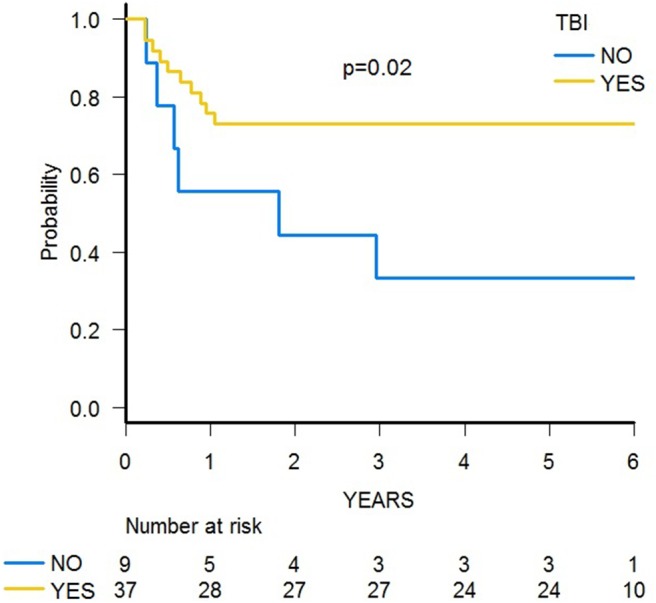
DFS probability of patients receiving a TBI-based or a chemo-based conditioning regimen.

With a median follow-up of 70.4 months (range 39.8–84.0), 31 patients are alive, and the 5-year probability of OS was 67.2% (95% CI 51.5–78.7) (Figure 5A). Since 14 patients relapsed, the 5-year probability of DFS was 65.2 (95% CI 49.6–77.0) (Supplementary Figure 3A). Five-year probability of GRFS was 60.9 (95% CI 45.3–73.3) (Figure 6A). As previously demonstrated in this setting (13), the use of a TBI-based conditioning resulted in an improved outcome. Indeed, DFS was 73.0% (95% CI 55.6–84.4) and 33.3% (95% CI 7.8–62.3) in patients who either did or did not receive TBI, respectively (*p* = 0.02; Figure 7); OS was 75.7% (95% CI 58.5–86.5) and 44.0% (95% CI 13.6–71.9) for these 2 groups (*p* = 0.10).

**Figure 8:**
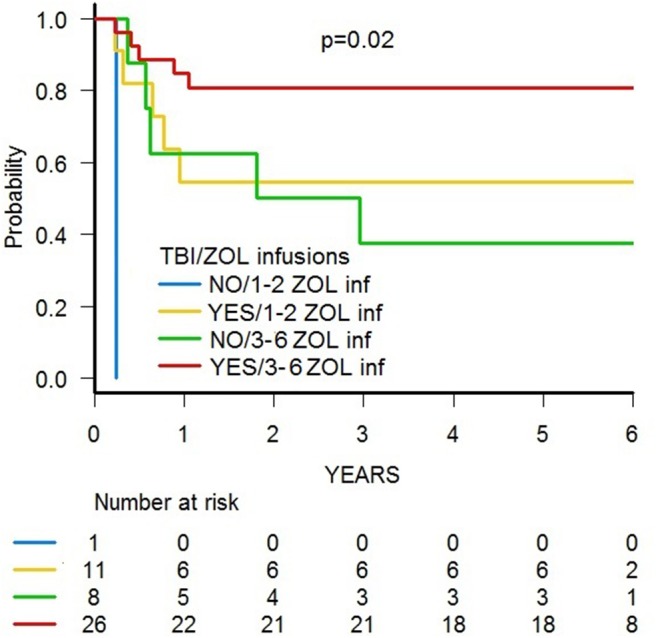
OS of patients stratified according to (i) the conditioning regimen used and (ii) the number of zoledronic acid (ZOL) infusion received after HSCT.

A trend toward higher survival estimates was observed in patients given 3 or more infusions as compared to those receiving 1–2 infusions, suggesting a possible influence of repeated administrations of zoledronic acid on outcome, although the difference was not statistically significant. In detail, OS was 73.1% (95% CI 54.7–85.1) for patients who received more than 2 infusions vs. 50% (95% CI 20.8–73.6) for those who received 1–2 infusions (*p* = 0.05) (Figure 5B), while DFS was 70.6% (95% CI 55.2–83.0) vs. 50.0% (95% CI 20.8–73.6) for patients given 3 or more infusions and those receiving 2 or less infusions, respectively (*p* = n.s.) (Supplementary Figure 3B). Notably, when stratified according to the conditioning regimen employed (TBI-based vs. chemo-based), the effect of repeated infusions of zoledronic acid was more evident, with the best OS obtained by patients who received both TBI and more than 2 zoledronic acid infusions (80.8%, 95% CI 59.8–91.5) (Figure 8). Moreover, in multivariable analysis model including these 2 variables, together with age at transplant and type of disease (ALL vs. AML), the repeated infusions of zoledronic acid showed an independent positive effect on OS (Table 2). GRFS differed significantly between patients receiving 1–2 infusion of zoledonic acid (33.3%, 95% CI 10.3–58.8) or ≥3 infusions (70.6%, 95% CI 52.2–83.0) (*p* = 0.006) (Figure 6B).

**Table 2:** OS multivariable analysis model.

**Variable**	**Hazard ratio**	**Lower 95% CI**	**Upper 95% CI**	***p*-value**
Use of TBI during conditioning regimen	0.277	0.074	1.035	0.056
Zoledronic Acid infusions > 2	0.273	0.082	0.905	0.033
Age at HSCT (< or > 10.9 years)	0.366	0.107	1.255	0.110
Disease (ALL vs. AML)	2.65	0.458	14.510	0.260

Outcome was not influenced by the number of TcRγδ cells infused with the graft (data not shown), nor by other factors, including age at transplant, type of leukemia (myeloid, T or B lineage, or MPAL), disease *status* at HSCT, NK alloreactivity, B-content score, or graft composition (data not shown).

## Discussion

Despite major improvements in T-cell depleted haploHSCT, disease relapse remains the main cause of treatment failure in acute leukemias (13); thus, strategies aimed at reducing leukemia recurrence are needed. TcRγδ T-lymphocytes are a subpopulation of T cells with features between innate and adaptive immunity endowed with notable properties (lysis of infected or stressed cells and cytokine and chemokine production among the others) through which they give unique contributions to immune reactions (33). Several studies have demonstrated the cytotoxic activity of TcRγδ T-cells against hematologic malignancies both *in vitro* (34) and *in vivo* (35), through direct recognition (i.e., in a MHC-independent way) of targets present on leukemia cells (36). Thus, not surprisingly, a faster immune reconstitution of this subset has been associated with an improved DFS after allogeneic HSCT in patients with either ALL or AML (9, 37, 38). In particular, seminal works by Lamb and colleagues in T-cell depleted HSCT from partially mismatched related donors demonstrated that accelerated TcRγδ T-cell recovery was associated with improved DFS (9, 37), mainly due to a decreased relapse incidence (39).

Kunzmann and colleagues first reported the activation and expansion of TcRγδ T-cells following the administration of aminobisphosphonates (17). The authors provided *in vitro* evidence that the proliferative response of TcRγδ T-cells to bisphosphonates was IL-2 dependent, whereas their activation occurred in the absence of cytokines (40). It has been shown that nitrogen-containing bisphosphonates, such as zoledronic acid, cause the inhibition of farnesyl diphosphate synthase in tumor cell lines, which leads to the accumulation of isopentenyl pyrophosphate (IPP), a type of non-peptidic-phosphorylated metabolite (nPAgs), which indirectly activates γδ T cell (41, 42). What still remains unclear is the precise mechanism by which TcRγδ T-cells are activated by nPAgs, although the γδ-TcR appears to undergo a conformational change or clustering of the butyrophilin family member 3A1 (BTN3A1) molecule (18). Although zoledronic acid increases the cytotoxic capacity of both principal subsets of TcRγδ T-cells (i.e., Vδ1 and Vδ2 (33)), Vδ2 T cells are more prone to this effect (20).

As detailed in recently published studies (12, 22), TcRαβ/CD19-depletion of G-CSF mobilized PBSC of haploidentical donors is an effective manipulation strategy, able to efficiently remove TcRαβ lymphocytes while retaining in the graft high numbers of effector cells, namely mature NK and TcRγδ T-cells. We characterized, by means of immunophenotypic and functional assays, the immune reconstitution of TcRγδ T-lymphocyte after this type of T-cell depleted haploHSCT (16), demonstrating that: i) TcRγδ T-cells are the predominant T-cell population in the early post-transplantation period, mainly deriving from cells infused with the graft; ii) these cells expand *in vivo* after transplantation; iii) they display a cytotoxic phenotype and degranulate when challenged with primary acute myeloid and lymphoid leukemia blasts; and iv) Vδ2 cells more efficiently lyse primary lymphoid and myeloid blasts *in vitro* after their exposure to zoledronic acid. Thus, this transplant platform constitutes the ideal setting to test the immune-modulatory properties of *in vivo* administration of aminobisphosphonates. Indeed, zoledronic acid has been previously demonstrated to be safe in pediatric age in studies on osteopenia (43) and, more importantly, at escalating doses (up to 4 mg/m^2^), in patients affected by recurrent/refractory neuroblastoma (44). Moreover, the use of bisphosphonates in HSCT recipients ameliorates osteoporosis, a well-known complication of transplantation (45). Indeed, the Australasian Leukemia and Lymphoma Group showed that post-transplant administration of zoledronate, based on a risk-adapted algorithm, minimizes bone loss (46).

We previously reported biological characterization, as well as short-term clinical data [median follow-up of surviving patients was 7.5 months (range 2.5–15) as compared to 70.4 months in the present study], of 43 patients receiving zoledronic acid after TcRαβ/CD19-depleted haploHSCT (20).

Our findings confirm that multiple infusions of zoledronic acid (up to 6 administrations) in children after HSCT, starting even a few weeks after the infusion of HSC, are well-tolerated and safe. In particular, no serious adverse event was recorded (specifically no cases of osteonecrosis of the jaw), as previously reported by Dieli in adults affected by prostate cancer (24) and by the aforementioned study of Russell in neuroblastoma patients (44). Moreover, the drug was safely administered in an outpatient setting, even in children aged 1 year or less.

As already reported (13), despite the lack of any pharmacological post-transplant prophylaxis, in our study the incidence of both acute and chronic GvHD was limited (with no patient experiencing grade III-IV acute or extensive chronic GvHD), much lower than that of other similar case series (47) or other type of graft manipulation, such as CD3/CD19-depletion (48). Of note, none of the patients experienced *de novo* onset or worsening of previously developed aGvHD, supporting the hypothesis that γδ T-lymphocytes do not cause GvHD (34). Moreover, our findings showing that patients receiving multiple infusions of zoledronic acid have a reduced incidence of both acute and chronic GvHD are of particular interest. Indeed, this is the first experience (20) of such an induced effect *in vivo* and deserves further investigation. Immunoregulatory properties of TcRγδ T-cells were first described by Patel and colleagues (49) and are now beginning to be investigated. Notably, Drobyski and colleagues noted that, in mice, activated TcRγδ T-cells are capable of modulating the ability of MHC-incompatible TcRαβ T-cells to cause GvHD after HSCT (50). Both Vδ1 and Vδ2 subsets may display regulatory properties, depending on different settings. Moreover, stimulation with pyrophosphates can induce regulatory capacities on Vδ2 T cells a few days after initial stimulation (51). In particular, IPP-stimulated Vδ2 T lymphocytes can inhibit the proliferation of CD4+ and CD8+ αβ T cells in response to strong recall antigens (52). Interestingly, strong co-stimulatory antigen-processing cell (APC) signals (i.e., acute GvHD initiating condition) seem to play an important role in the induction of these suppressive properties. The mechanisms of suppression by TcRγδ T-cells are still not clear; while some authors identified TGFβ, IL-10, and other suppressive cytokines as mediators of these effects (53), other data suggested that cell-to-cell interactions, via CD80, CD86, and PDL-1 expressed on Vδ2 T lymphocytes, are necessary to achieve suppression of other cell populations (54). Another unresolved issue is the lack of defined regulatory γδ T-cell phenotype (51), although CD39 (55) and latency-associated peptide (a membrane-bound TGF-β1) (56) have been identified as putative markers; thus, further studies are needed to address this question.

The cumulative incidence of TRM in our study cohort was remarkably low (4.3%), with 2 patients dying due to infection. Repeated infusions of zoledronic acid seems to also have a role in reducing TRM, since we recorded no infectious deaths in children who received more than 2 administrations. The well-known anti-infectious role of TcRγδ T-cells has been very recently demonstrated by Perko and colleagues in children after HSCT (38). Indeed, using a logistic regression model, they showed that the higher the number of TcRγδ T-cells after HSCT, the lower the risk of infection, which results in an improved EFS. However, because of the limited number of patients per cohort, subanalysis on different types of donors (siblings, MUDs, haploidentical relatives, and umbilical cord blood) failed to demonstrate a similar effect in any single subgroup. In our study, repeated infusions of zoledronic acid did not affect the incidence of infections of either viral, bacterial, or fungal origin. However, patients receiving multiple infusions had a reduced, although not statistically significant, cumulative incidence of CMV infection. Given the low number of patients enrolled in this study, it is possible that the number of events was not sufficient to highlight such an effect.

The OS estimate observed in our study (without any difference between ALL, either of T or B lineage, AML patients), in line with our recently published cohort (13), is higher than that reported in other pediatric trials of CD34 selected (57, 58) or CD3/CD19-depleted haploidentical HSCT (48), suggesting a favorable impact of TcRγδ T-cells on outcome. Outcome data were also similar to those reported by Maschan et al., who used the same type of graft manipulation, although with different study population and donors (47), making comparison difficult to interpret. Smetak and colleagues studied a different method of T-cell depletion, namely based on CD4+ and CD8+ depletion, able to leave in the graft innate lymphocytes such as TcRγδ T cells and NK cells (59). Despite similar depletion capacity, other authors noted that the B-cell content of the graft was high, raising concerns about the risk of EBV-associated PTLD (60).

We previously demonstrated that the use of TBI in the conditioning regimen positively influenced the outcome in this setting, probably due to a potent antileukemia effect, compensating for the lack of TcRαβ cell–mediated GvL effect (13). Here, we show that patients receiving more than 2 infusions of zoledronic acid seem to have an improved OS, although the difference did not reach the statistical significance level. However, when stratified according to the type of conditioning regimen employed, the independent beneficial effect of zoledronic acid on OS became evident, as also highlighted by multivariable analysis. As zoledronic acid improves cytotoxicity of TcRγδ T-cells, the initial hypothesis was that multiple infusions could reduce RI. However, due to the limited number of patients enrolled and events observed, it was not possible to draw any firm conclusion; in particular, we were not able to highlight a significant reduction of RI. Since time to relapse and disease status were comparable between patients receiving 1–2 or 3–6 infusions of zoledronic acid, the trend toward a better outcome of patients receiving multiple infusions does not seem to be due to the fact that those patients had longer DFS allowing for multiple infusions nor because they had a less aggressive disease.

We were not able to demonstrate an association between an increased number of TcRγδ T-cells after HSCT and an improved outcome (data not shown), as demonstrated by Lamb *et al*. (9, 37). A possible explanation for these apparently discordant findings can be given by the observation that, as shown by our data, the percentage and absolute number of TcRγδ T-lymphocytes, and especially of the Vδ2 subset, decrease over time after prolonged exposure to zoledronic acid (20). This phenomenon, in contrast with the first *in vitro* observations by Kunzmann (40), was previously proven by Dieli and coauthors in patients treated with zoledronic acid for hormone-refractory prostate cancer (24) and by Kalyan and colleagues in post-menopausal women treated with bisphosphonates for osteoporosis (61). These authors demonstrated an inhibitory effect on TcRγδ T-cells produced by neutrophils upon bisphosphonate uptake through the production of reactive oxygen species (62). Although the use of IL-2 could improve proliferation of TcRγδ T-lymphocytes (24), we decided not to administer this cytokine because of a possible increase in the risk of GvHD (63).

The main limitations of this study include: i) the lack of a control arm and ii) a variability of the administration schedule, this limiting the possibility to draw firm conclusions on the number and timing of zoledronic acid administration. Finally, we did not systematically collect data on bone metabolism pre and post zoledronic acid infusions. Since it is well-known that patients undergoing HSCT suffer from osteopenia (64), data on bone metabolism after zoledronic acid administration could help guide post-transplant supportive therapies.

In conclusion, our data indicate that the infusion of zoledronic acid after TcRαβ/CD19-depleted haploidentical HSCT is safe. Three or more infusions of zoledronic acid result in a lower incidence of both acute and chronic GvHD and lower TRM. Moreover, they had an independent effect in ameliorating the outcome. Further investigation (a non-randomized prospective trial is currently ongoing at Wisconsin University, ClinicalTrials.gov Identifier: NCT02508038), namely a randomized-controlled trial, is needed to confirm these data.

## Data Availability Statement

The datasets generated for this study are available on request to the corresponding author.

## Ethics Statement

The study involving human participants was reviewed and approved by the Ethical Committee of Bambino Gesù Children's Hospital. Written informed consent to participate in this study was provided by the participants' legal guardian/next of kin.

## Author Contributions

PM, MA, FG, GM, GP, LS, DP, and FL designed the study, analyzed data, and wrote the paper. VB, SB, EG, MS, and IA performed graft manipulation, characterized the graft, and edited the paper. PM, MA, FG, GM, MB, GL, EB, LG, SG, DP, and FL treated patients, collected data, analyzed data, and edited the paper. All authors approved the final version of the paper.

### Conflict of Interest

The authors declare that the research was conducted in the absence of any commercial or financial relationships that could be construed as a potential conflict of interest.

## Supplementary Material

Supplementary Figure 1 Cumulative incidence of relapse and TRM of the whole cohort of 46 patients.

Supplementary Figure 2 Cumulative incidence of relapse of patients receiving either 1–3 or 4–6 infusions of zoledronic acid (ZOL).

Supplementary Figure 3 DFS of the whole cohort of 46 patients. B. DFS of patients receiving either 1-2 or 3-6 infusions of zoledronic acid (ZOL).
