# Supplementary material for: Immune Modulation Properties of Zoledronic Acid on TcRγδ T-Lymphocytes After TcRαβ/CD19-Depleted Haploidentical Stem Cell Transplantation: An analysis on 46 Pediatric Patients Affected by Acute Leukemia
Source: Front Immunol. 2020 May 12;11:699. doi: 10.3389/fimmu.2020.00699 (PMC7235359; doi:10.3389/fimmu.2020.00699)
Supplement: Supplementary file 1 [file Data_Sheet_1.docx]

**Supplementary material**

**Conditioning regimens**

Among the 46 enrolled patients, the conditioning regimen consisted of Total Body Irradiation (TBI) (200 cGy twice daily for 3 consecutive days), Thiotepa (10 mg/kg, administered in 2 divided doses) and Fludarabine (40 mg/m2/day, administered for 4 consecutive days) for 19 children (41%); TBI (200 cGy twice daily for 3 consecutive days), Thiotepa (10 mg/kg, administered in 2 divided doses) and Melphalan (140 mg/m2) for 12 patients (26%); TBI (200 cGy twice daily for 3 consecutive days) and Melphalan (140 mg/m2) for 4 patients (9%); Busulfan (dose per kg according to manufacturer’s indications administered in 16 divided doses, through 4 days, and adjusted on the basis of first-dose pharmacokinetic analysis, in order to obtain a steady-state concentration of 600-900 ng/ml), Thiotepa (10 mg/kg, administered in 2 divided doses) and Fludarabine (40 mg/m2/day administered for 4 consecutive days) for 4 patients (9%); Busulfan, Cyclophosphamide (60 mg/kg/day for 2 consecutive days) and Melphalan (140 mg/m2) for 4 patients (9%); other conditioning for 3 patients (6%).
