# Supplementary figures and images for: Immune Modulation Properties of Zoledronic Acid on TcRγδ T-Lymphocytes After TcRαβ/CD19-Depleted Haploidentical Stem Cell Transplantation: An analysis on 46 Pediatric Patients Affected by Acute Leukemia
Source: Front Immunol. 2020 May 12;11:699. doi: 10.3389/fimmu.2020.00699 (PMC7235359; doi:10.3389/fimmu.2020.00699)

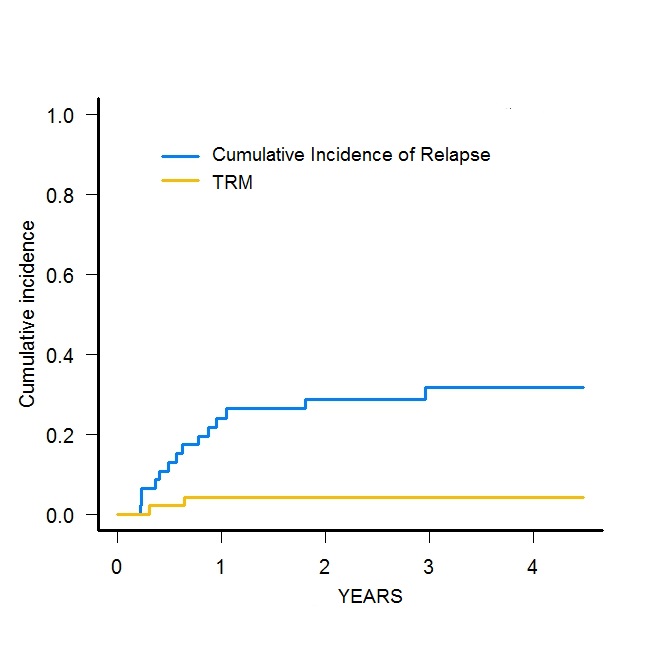

Supplement: Supplementary Figure 1 — Cumulative incidence of relapse and TRM of the whole cohort of 46 patients. [file Image_1.jpeg]

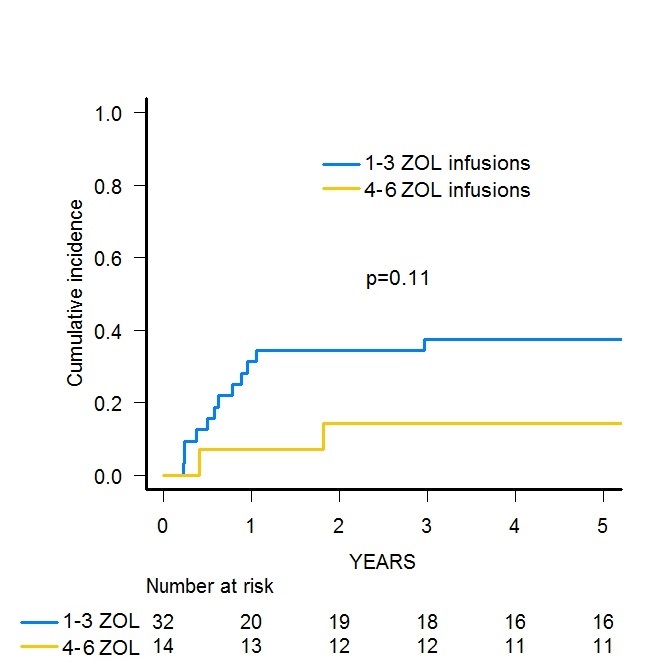

Supplement: Supplementary Figure 2 — Cumulative incidence of relapse of patients receiving either 1–3 or 4–6 infusions of zoledronic acid (ZOL). [file Image_2.jpeg]

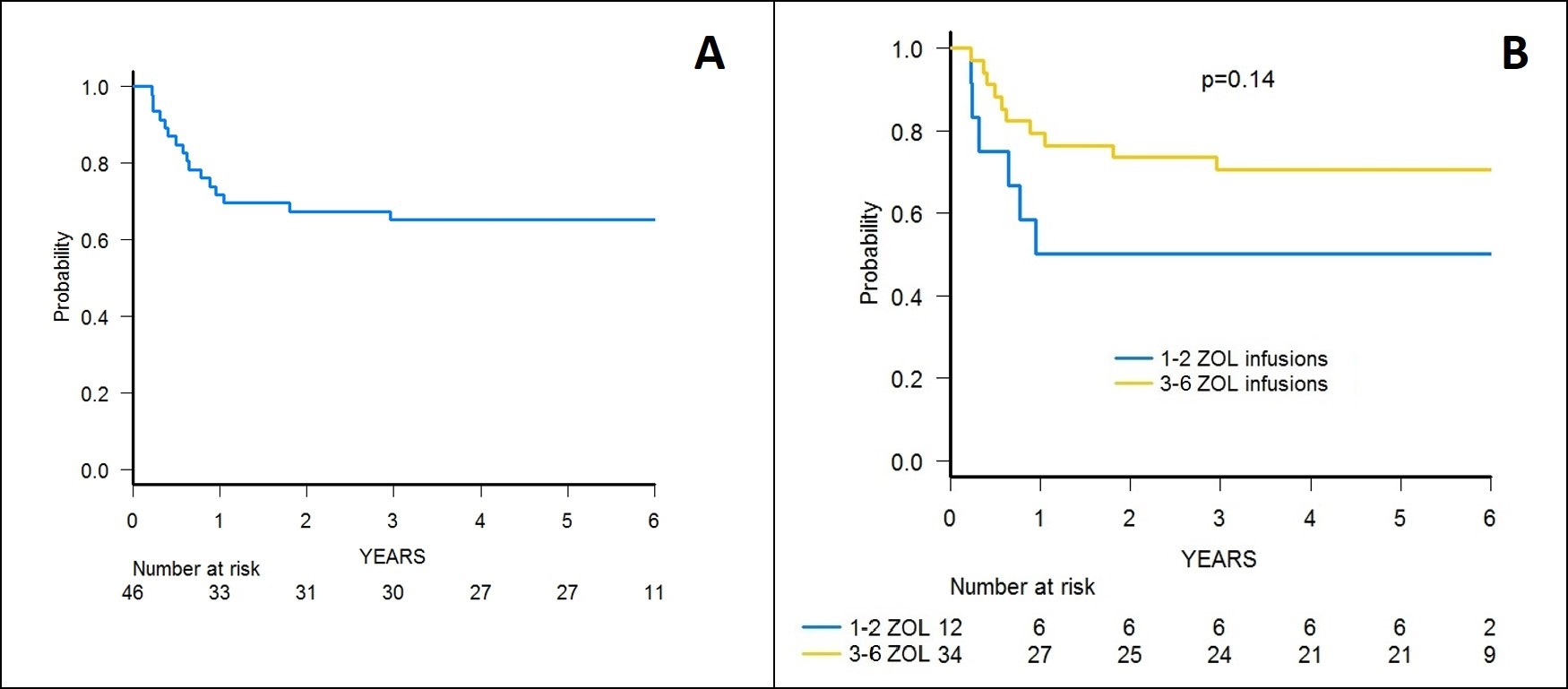

Supplement: Supplementary Figure 3 — DFS of the whole cohort of 46 patients. B. DFS of patients receiving either 1-2 or 3-6 infusions of zoledronic acid (ZOL). [file Image_3.jpeg]
